# Effectiveness of clinical integrated short course training of evidence based medicine for postgraduate medical specialty students in Ethiopia in 2022: A randomized control trial

**DOI:** 10.1371/journal.pone.0277886

**Published:** 2023-01-20

**Authors:** Atalel Fentahun Awedew, Eyob Girma Abera

**Affiliations:** 1 Department of Surgery, Addis Ababa University, Addis Ababa, Ethiopia; 2 Department of Public Health, Jimma University, Jimma, Oromia, Ethiopia; AIIMS: All India Institute of Medical Sciences, INDIA

## Abstract

**Background:**

Evidence based medicine (EBM) is a newly emerged philosophy of medical education and health care service which brings quality of health service, efficient use of materials, and patient satisfaction.

**Objective:**

To investigate the effectiveness of clinical integrated short course training of EBM for post-graduation medical specialty students in Ethiopia, 2022.

**Method:**

Randomized, single blind, superiority trial, and multi-centric experimental study design employed. The eligible candidates randomly assigned to the interventional group and the control group (waitlisted). The allocation of the participant concealed from the principal investigator and participant. Sample size was determined with a two-sided test and α level of 5% and 80% power with the total of 52 calculated sample size and 44 (21 for intervention and 23 for control group) students were used for final analysis. Mann Whitney U test and Independent Sample T test used to test mean difference between intervention and control group after checking normality distributions to estimates the amount by which the training changes the outcome on average compared with the control. The result of the final model expressed in terms of adjusted mean difference and 95% CI; statistical significance declared if the P-value is less than 0.05.

**Results:**

Among 44 postgraduate students, only 29.5% practices EBM during delivering of clinical services. Overall EBM knowledge was changed with adjusted mean difference (Mean±SD 17.55 (13.9, 21.3), p<000). This training provided significant change in all main domain of EBM, more in validity evaluation of the study (Mean±SD, 3.8(1.3, 6.2), p<0.0018) and impact of study design (Mean±SD, 3.8(2.6, 5.1), p<0.000). There was also significant change of overall attitude with adjusted mean difference (Mean±SD, -8.2(-9.6,-6.7), p = 000).

**Conclusion:**

Clinical integrated EBM training brought significant change of knowledge and skills of principles and foundations of EBM. Adopting principles of EBM into curricula of postgraduate specialty students would assure the quality of medical care and educations.

## Introduction

Evidence based medicine (EBM) is a newly emerged philosophy of medical education and health care service. It is the process of systematically, planned, and comprehensive searching, selecting, reviewing of clinical researching findings, critical appraising, customizing for clinical practice conjugated with previous clinical practice and patients value and expectations [1, 2]. EBM is a fundamental and new medical educational philosophical method [2, 3] to achieve quality of clinical core competency of medical graduates such as scientific foundation of medicine, clinical skills, population health and health systems, communication skills, professionalism, management of information, critical thinking, practice-based improvement and research and inter-professional collaborative practice. In the view of clinical sphere, best external evidence, patients’ expectation and values, and clinical expertise have been a central dogma (triads) for implication of EBM in clinical practice to diagnosis, treat, predict prognosis, and prevent disease [4, 5]. EBM gets a great attention globally and Institute of Medicine’s Roundtable on Evidence-Based Medicine set a goal which stated that more than 90% of clinical decision will be supported by accurate, timely and up-to-date clinical information and will reflect the best available evidence to achieve the best patient outcomes in 2020 [6]. EBM practice has five basic steps such as developing well-built answerable question, systematic searching of the evidence, critical appraisal and graded of the evidence, apply the evidence to make decision with integration of previous clinical experience and patients expectation and values, and efficacy evaluation of this application of evidence on a patient and areas for improvement [7, 8]. Each steps of EBM practice needs its own knowledge and skills which will obtain from different level trainings. Currently, EBM has incorporated in undergraduates and postgraduates medical training programs [4] in developed countries. More than 70% of health care provider in developed countries such as UK [9], Croatia [10], China [11], Japan [12], and Italy [13] have a good practicing of EBM in clinical making decisions to improve quality of care, patient satisfaction, and efficient use of resources. In some developed countries such as Iran [14], Israel [15], Norway [16], and France [17] have low level of practicing EBM in the clinical care delivery. However, practicing of EBM in medical teaching and clinical practice has substantially limited in developing countries [18, 19]. Evidences from different survey confirmed that there was low knowledge and practice of EBM in health care provider centers and academic institution in most of developing countries, particularly in Africa [18–21]. In Sudan, only 10% of medical doctors have been utilized EBM in their 50–100% of clinical practice to deliver quality health care [22]. A study conducted on health care provider and academician in higher education institutions in Namibia, Mozambique, Lesotho and Botswana revealed that more than 74% of higher academic institution health care provider had poor knowledge and practice of evidence of medicine [20], despite of, cochrane center was established in South Africa since 2015 [23]. A study conducted in Egypt physicians reported that approximately 11% of physicians have good knowledge of principles, terms, skills, critical appraisal and application of EBM [24]. According to a study conducted in Ethiopian revealed that less than 32% [19] to 48% [18] of medical professionals were practicing EBM to deliver quality clinical service. Implementation of EBM brings competent and global health professionals, quality and standard of health care, and efficient and effective use of resource [25]. From five professors from UK, New Zealand, Canada, and Australia have strongly recommended that EBP curriculum considerations, teaching evidence based practice (EBP), and stakeholder engagement in evidence based practice (EBP) education are a three key determinate to develop culture of EBM among healthcare provider [26]. Other evidences from many global randomized control trials in different countries revealed that training of EBM had shown improvements of knowledge, attitude, and skill of EBM [27–30]. However, evidence on effectiveness of training on EBM in Africa and specifically in Ethiopia is scarce. Therefore it is the right time to determine the effectiveness of clinical integrated training of EBM to improve attitude, knowledge and practice of EBM. The findings will have an input for policy makers to plan and incorporate EBM to medical curricula. It also will be important input for clinical practice, patients, and researchers.

## Methods

### Study area and period

The study was conducted in Ethiopia within 22 postgraduate medical specialties that has been given in different medical schools [31]. The study was conducted from April 1-June 25, 2022.

### Study design

Randomized, single blind, superiority trail, and multi-centric experimental study design were employed. The principal investigator invited all postgraduate medical specialties from different postgraduates’ schools through telegram group and email and all interested candidates registered with specific code. Then, eligibility of the participants was screened. Once the participants screened for eligibility, independent person randomly allocated the candidate into interventional group and waitlisted the control group. The allocation of the participant concealed from the principal investigator and participants, and the training delivered for intervention group before the control group. The training was adopted from previous validated and delivered courses [27, 28, 32]. It prepared using theory of adult learning [33, 34], educational strategy [34–36], and reviewing from previous effective EBM training program [37]. Interactive lecturing, problem based Learning (PBL), case based discussion (CBD), and practical sessions were mode of teaching. The intervention delivered through E-learning mode of teaching that was 4 hrs per week for 4weeks.

#### Population

*Source of population*. Postgraduate medical specialty registered in different university health science schools, faculty, and colleges in Ethiopia.

*Study population*. The intervention groups were all postgraduate students who have taken more than 80% of the training (those who attended the courses a minimum of three weeks for four hours), and the control groups (waitlisted) were randomly selected postgraduate students from different schools.

#### Eligibility criteria

*Inclusion criteria*. Volunteer postgraduate medical students (residents) who have taken more than three week of the training course.

*Exclusion criteria*. Those who have taken less three weeks of the training course, medical interns and specialists.

*Sample size*. Sample size determined with a two-sided test and α level of 5% and 80% power. We assumed that the equivalence margin is chosen to be 3% (i.e., δ = 0.03). Also, suppose the true difference in mean of overall knowledge 10% (i.e., d = 0.1) and the standard deviation of 10% (i.e., SD = 0.1). For achieving an 80% power (i.e., 1−β = 0.8) at the 5% level of significance (i.e., α = 0.05) with intervention to control group ration (k = 1), the total sample size was 52 (26 for intervention and 26 for the control group). The sample size was calculated with online sample size calculator (https://riskcalc.org/samplesize/) [38, 39].

#### Sampling technique

The principal investigator invited for all accessible postgraduate medical students and all registered trainee received an identification code. Then, eligibility of the participants screened. Once the participants screened for eligibility, independent person randomly allocated the candidate into interventional group and control group (waitlisted from the training). The allocation of the participant was concealed from the principal investigator and participants.

### Data collection tools and procedure

Data collection tool was adopted from standard tools [40, 41] and reviewing different literature [28]. The Fresno test is a well-accepted instrument to evaluate four steeps of EBM [40–42]. Outcomes of the training were measured with framework of EBM competency such as attitude, knowledge, and skill [43]. Competency of EBM such as attitude, knowledge, and skill measured with modified previous validated tools [40, 41] and published literatures [28]. The tool was pretested with 5% of the sample.

### Data analysis and processing

All the self-administered questionnaires were checked manually for completeness and consistency. The collected data coded and entered to SPSS version 24, STATA 14, and RevMan5.3 for data processing and analysis. Simple descriptive statistics such as a frequency distribution and percentages performed to describe the demographic and socioeconomic. Mann Whitney U test and Independent Sample T test were used to test mean difference between intervention and control group after checking normality distributions. The result of the final model expressed in terms of adjusted mean difference and 95% CI and statistical significance was declared if the P-value is less than 0.05.

### Data quality control

The data collectors were residents and they trained for one day. The principal investigator had an ongoing supervision and reviewing of each completed data during the data collection to ensure the quality of data by checking filled formats for their completeness and consistency thought out the data collection period. Contamination of training materials and trainee between intervention and control group secured. Data collection instrument pretested by 5% of sample size before data collection, then any errors or ambiguity on the questionnaire were corrected and modified.

### Ethical consideration

Ethical clearance was obtained from Addis Ababa Medical and Business College Institutional Review Board. Data collection was started after approval and obtained oral informed consent from the participants. All information obtained from participant kept confidentially.

## Results

**Fig 1:**
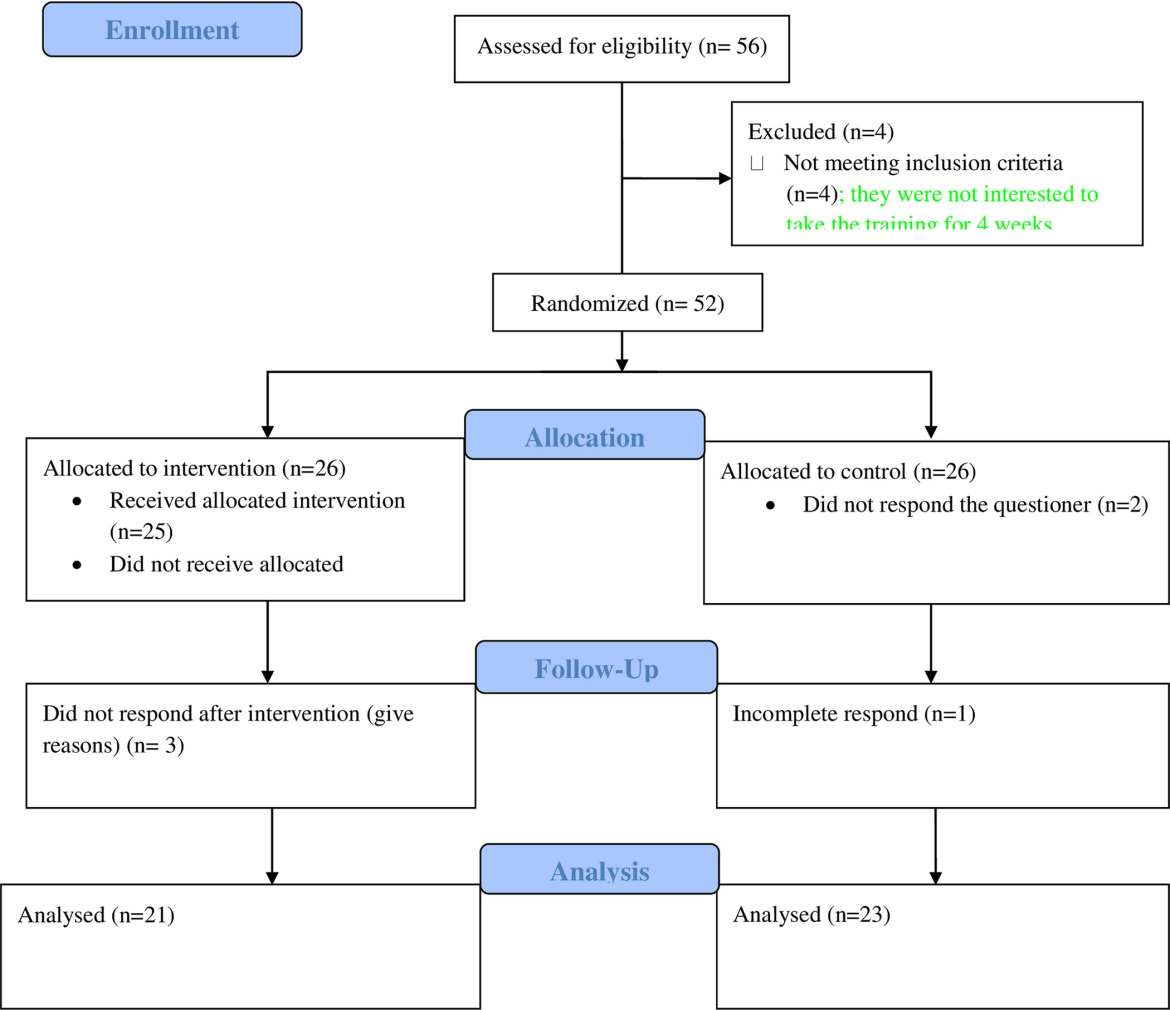
Consort flow chart of the study participant selection.

Fifty two eligible postgraduate specialty students were interested to take EBM training from different universities. They were randomized to intervention group (26 students) and waitlist control group (26 students). Data were collected from the control group at first and training was given for the intervention group. Finally, the training was also provided for the control group. Of 52 eligible and randomly assigned students, 44 students were used for final analysis (21 students from intervention and 23 control group) (Fig 1).

Both control and intervention group were predominantly males (65.2%, 61.9%) with almost similar mean age of both groups (29.6 years, 29.3years) respectively. More than half (52%) of control group had history of research training while only 38% of intervention group have taken research trainings. However, both the control and intervention group didn’t take EBM training (Table 1). Among 44 postgraduate students, only 13 (29.5%) practices EBM during delivering of clinical services-18.2% of students practice less than 25% of their clinical practice and 11.4% of students practice 25–50% of their clinical practice.

**Table 1:** Baseline characteristics of the study post graduate specialty in Ethiopia 2022.

Characteristics		Intervention group	Control group
		(N = 21)	(N = 23)
Age	Mean age	29.6(2.6)	29.3(2.8)
Sex	Male	14(66.9%)	14(60.9%)
Specialty	Surgery	9(42.9%)	9(39.1%)
	internal medicine	4(19%)	5(21.7%)
	pediatrics	3(14.3%)	4(17.4%)
	Orthopedics	3(14.3%)	3(13.0%)
	Oncology	2(9.6%)	1(4.3%)
	Psychiatry	0(0%)	1(4.3%)
Level of residency			
	R1	5(23.8%)	3(13%)
	R2	4(19%)	7(30.4%)
	R3	8(38.1%)	6(26.1%)
	R4	4(19%)	7(30.4%)
Experience working as GP	1year and below	10 (47.6%)	14(60.9%)
	2year	7(33.3%)	6(26.1%)
	3year	2(9.5%)	2(8.7%)
	above 3	2(9.5%)	1(4.3%)
Research Training	Yes	8(38.1%)	12(52.2%)
EBM training	No	21(100%)	23(100%)
Publishing Research	No	2(9.5%)	2(8.7%)
Publishing Research(PI)	No	21(100%)	0
Publishing Research(Co-author)	No	2(9.5%)	2(8.7%)
Practice EBM	Yes = 13	6(28.6%)	7(30.4%)

This study found that EBM training brought significant change in all main domain of EBM. The overall EBM knowledge was changed with adjusted mean difference (Mean±SD 17.55 (13.9, 21.3), p<000). This training provided significant change in all main domain of EBM, more in validity evaluation of the study (M Mean±SD, 3.8 (1.3, 6.2), p<0.0018) and impact of study design (Mean±SD, 3.8(2.6, 5.1), p<0.000) (Table 2).

**Table 2:** Change of knowledge after training in postgraduate specialty students in Ethiopia, 2022.

Domain	Intervention group (N = 21)	Control group (N = 23)	Mean difference(95%CI)	P value
	(Mean±SD)	(Mean±SD)		
Question development	5.5(0.9)	3.4(1.3)	2.13(1.4, 2.8)	<000
Source identification	4.9(0.98)	3.6(1.0)	1.3 (0.7, 1.9)	<0.001
Study design	9.7(1.9)	5.9(2.3)	3.8(2.6, 5.1)	<000
Searching and limit	6.8(1.0)	4.6(1.5)	2.15(1.4, 2.9)	<000
Relevance	9.6(2.0)	7.4(1.9)	2.2(0.9, 3.4)	0.0005
Validity	20.1(3.8)	16.2(4.4)	3.8(1.3, 6.2)	<0018
Effect size	9.6(2.1)	7.4(1.9)	2.2(0.9, 3.4)	0.005
Overall knowledge	66.4(4.4)	48.4(7.1)	17.55(13.9, 21.3)	<000

There was also significant change of overall attitude with adjusted mean difference (Mean±SD, -8.2 (-9.6,-6.7), p = 000) (Table 3).

**Table 3:** Change of attitude after training among postgraduate specialty students in Ethiopia, 2022.

Domain	Intervention group (N = 21)	Control group (N = 23)	Mean difference(95%CI)	P value
	(mean±SD)	(mean±SD)		
Original research is confusing	4(0.9)	2.8(0.83)	1.22(0.68, 1.70)	<000
Study design is important in article selection	2.2(1.4)	2.7(1.0)	-0.55(-1.3, 0.4)	0.92
Evidence-based decision making is ’health care by numbers’	4.3(0.7)	2.7(0.9)	1.6(1.08, 2.1)	0.001
Contracts for health care professionals should include time taken away from patient care for reading and appraising the literature	4.2(0.60)	2.8(0.9)	1.4(0.8, 1.8)	0.000
I am confident that I can assess research evidence	1.7(0.6)	2.5(1.2)	-0.85(-1.4, -0.3)	0.98
Systematic reviews play a key role in informing evidence-based decision making	1.6(0.9)	2.4(1.0)	-0.88(-1.4, -0.05)	0.98
The health care system in my country should have its own programme of research about clinical effectiveness	3.3(1.3)	3.0(1.01)	0.3(-0.4, 1.3)	0.20
I need to incorporate EBM in medical curriculum	1.5(1.2)	2.7(1.1)	-1.3(-1.8, -0.71)	.000
I strongly believed that EBM improves quality of health care and decreased health care cost	1.7(0.8)	2.2(0.98)	-0.45(-1.00, 0.08)	0.95
EBM is the fundamental tools to achieve quality medical education, continuous professional development, and lifelong learning	1.2 (0.4)	2.3(0.8)	-1.1 (-1.5, -0.7)	0.998
Meta-analysis of RCT with low risk is the highest quality of Evidence	1.3(0.6)	2.7(0.8)	-1.5(-1.8, -0.98)	.000
I am confident in searching of best external evidence in different search electronic data base	1.5(0.7)	2.4(1.2)	-0.82(-1.4, -0.21)	0.98
I am confident in developing of question(PICOS, PIRDS, CoPop…) to find best external evidence in different search electronic data base	1.8(0.7)	4(1.1)	-2.2(-2.8, -1.6)	0.000
I am confident and familiar to AMSTAR II, CONSORT, Newcastle-Ottawa Scale, JBI quality tools, and cochrane RCT tools to critical appraisal of evidences	1.8(0.8)	3.5(1.1)	-1.8(-2.3, -1.2)	0.000
I am confidence to GRADE evidence	2(0.6)	3.4(1.1)	-1.4(-1.9, 0.85)	0.000
Overall attitude score	34.1(3.3)	42.3(5.3)	-8.2(-9.6, -6.7)	0.000

1 = strongly agree, 2 = agree, 3 = neither agree nor disagree, 4 = disagree, 5 = strongly disagree.

## Discussion

We found that there was low practice of EBM during deliver of clinical care service. This finding was consistent with previous Ethiopian studies [18, 19] as well as most developing countries [22]. According to a study conducted in Ethiopian revealed that less than 32% to 48% of medical professionals were practicing EBM to deliver quality clinical service [18, 19]. Low practice of EBM practice observed in developing countries such as Sudan [22], Jordan, and Egypt. This study found that clinical integrated EBM training would provide significant benefit to improve basic principles and foundation of EBM.

The study noted that clinical integrated EBM training improved question development skills, source identification and evaluation, study design selection, searching and limit, relevance, validity of the study, effect size and overall knowledge and skills on EBM. The training also had effect on changing people’s attitudes toward evidence-based medicine. Its findings were consistent with previous findings from around the world [27, 28, 32]. An international cluster randomized control trial among Obstetrics and Gynecology postgraduate students in Argentina, Brazil, the Democratic Republic of the Congo, India, the Philippines, South Africa, and Thailand from 2009 to 2010 found that clinical integrated EBM training improved knowledge, skill, and educational environment [28]. Another research finding from five European countries stated that harmonized EBM training resulted in a significant change in knowledge and skills of EBM principles [32]. Most medical school in developed countries incorporated in their undergraduates and postgraduate training programs [44–46]. The Accreditation Council for Graduate Medical Education and the Association of American Medical Colleges have also recommended that biostatistics, clinical epidemiology, medical informatics, and evidence-based medicine skills be introduced into both undergraduate and postgraduate medical curricula [46]. Developed countries such as UK [9], Croatia [10], China [11], Japan [12], and Italy [13] have good practicing of EBM in clinical making decisions to improve quality of care, patient satisfaction, and efficient use of resources.

**Fig 2:**
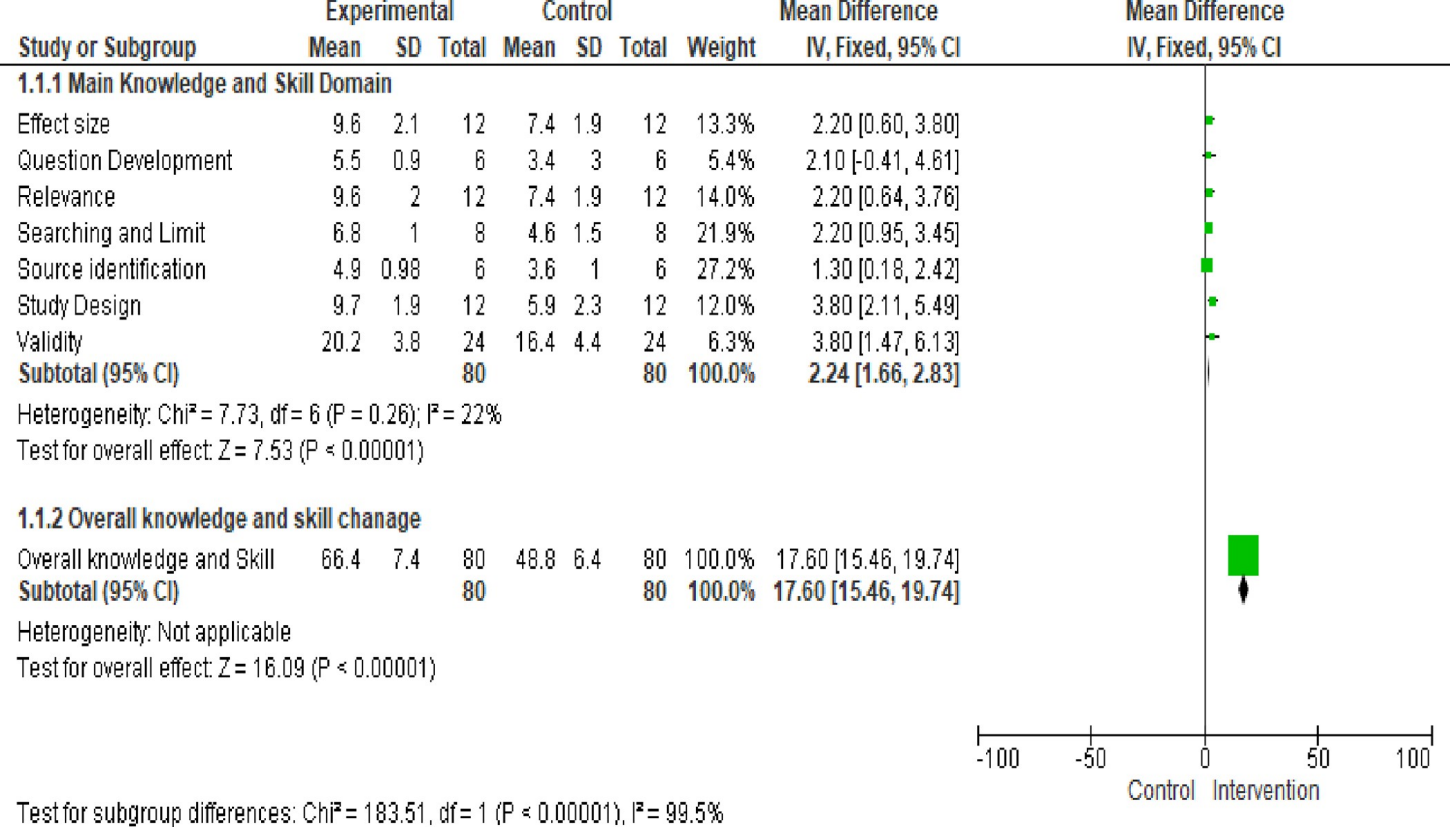
Frost plot of change of main domain of knowledge and skill for post graduate students in Ethiopia 2022.

**Fig 3:**
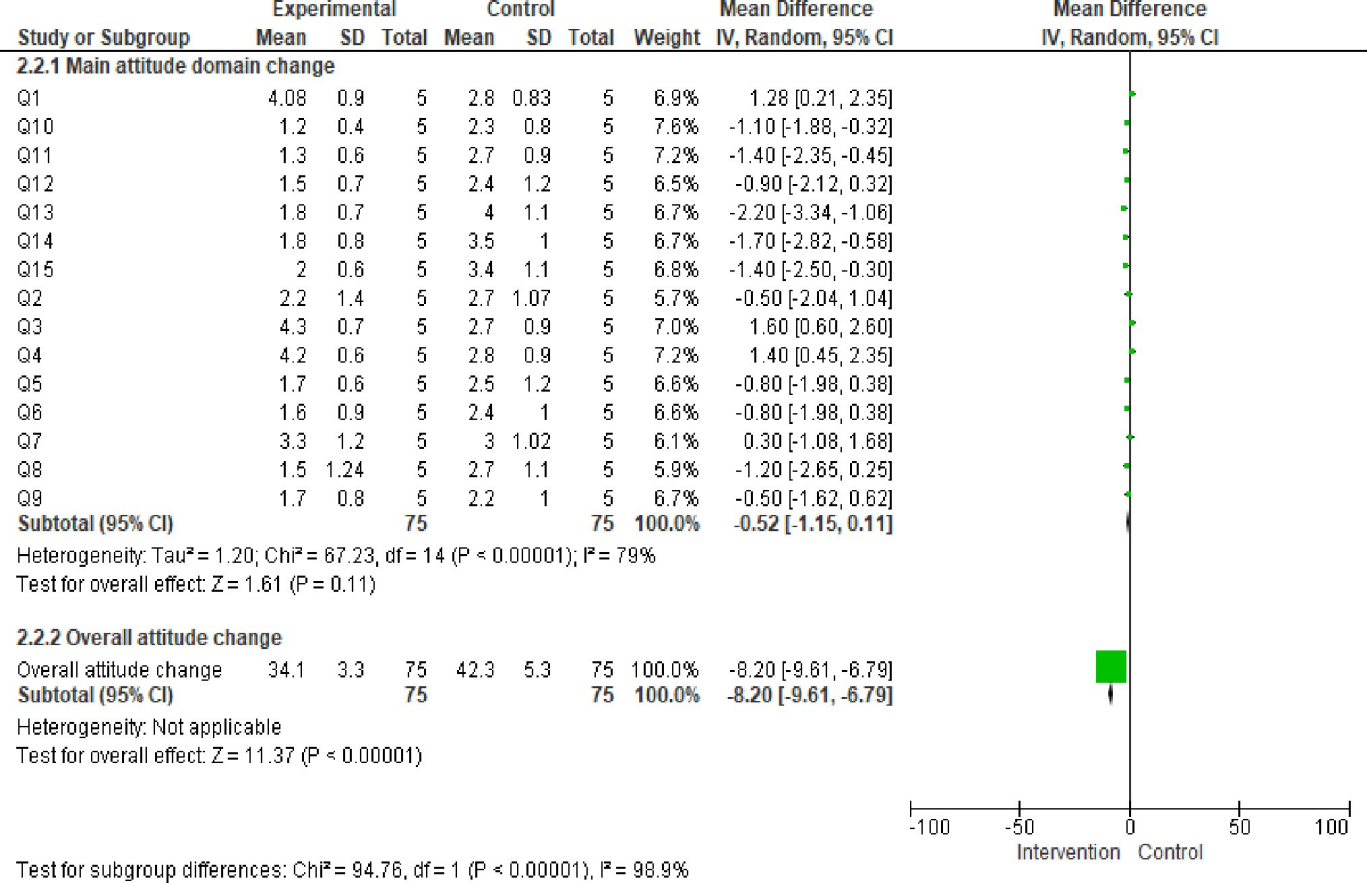
Frost plot of change of main domain of attitude of post graduate students in Ethiopia 2022.

However, knowledge, skill, and practice of EBM are low in developing countries. A study conducted in Jordan on family physicians revealed that level of EBM practices were less than 40% of physicians in their clinical practice [47]. In Sudan, only 10% of medical doctors have been utilized EBM in their 50–100% of clinical practice to deliver quality health care [22]. A study conducted in Egypt physicians reported that more than 76% have good attitude towards EBM; however, only approximately 11% of physicians have good knowledge of principles, terms, skills, critical appraisal and application of EBM [24]. According to a study conducted in Ethiopian revealed that less than 32% [19] to 48% [18] of medical professionals were practicing EBM to deliver quality clinical service. Experts recommended that incorporate of EBM in medical curriculum, teaching evidence based practice (EBP), and stakeholder engagement in evidence based practice (EBP) education are a three key determinate to improve EBM in health care provider [48]. This study found that EBM training brought significant change in all main domain of EBM (Figs 2 & 3).

As a result, this study provides a path to improved evidence-based medicine skills and knowledge for Ethiopia as well as developing countries, particularly in Africa. The three pillars of EBM recommendation from five professors from the UK, New Zealand, Canada, and Australia-EBP curriculum considerations, teaching evidence based practice (EBP), and stakeholder engagement in evidence based practice (EBP) education were the foundation of EBM curricula and training in most developed countries [26].

## Conclusion

Clinical integrated EBM training brought significant change of knowledge and skills of principles and foundations of EBM. Adopting principles of EBM into curricula of postgraduate specialty students would assure the quality of medical care and educations.

## Abbreviation

EBM: Evidence Based Medicine
MOH: Ministry of Heath
MOSHE: Ministry of Science and Higher Education
OR: Odd Ratio
RR: Relative Risk
SD: Standard Deviation
UK: United Kingdom
